# Corrected Allele Frequency of BRCA1/2 Mutations Is an Independent Prognostic Factor for Treatment Response to PARP-Inhibitors in Ovarian Cancer Patients

**DOI:** 10.3390/jpm12091467

**Published:** 2022-09-07

**Authors:** Christina T. Grech, Dietmar Pils, Stefanie Aust, Christoph Grimm, Stephan Polterauer, Alexander Reinthaller, Leonhard Müllauer, Theresa Reischer, Christine Bekos

**Affiliations:** 1 Department of Obstetrics and Gynecology, Division of General Gynecology and Gynecologic Oncology, Gynecologic Cancer Unit, Comprehensive Cancer Center (CCC), Medical University of Vienna, 1090 Vienna, Austria; 2 Department of General Surgery, Division of Visceral Surgery, Comprehensive Cancer Center (CCC) Vienna, Medical University of Vienna, 1090 Vienna, Austria; 3 Department of Pathology, Medical University of Vienna, 1090 Vienna, Austria

**Keywords:** BRCA, PARP-inhibitor, ovarian cancer, allele frequency

## Abstract

PARP inhibitors (PARPi) have increased treatment options in ovarian cancer, particularly in patients with BRCA1/2 mutations, although there are still marked differences in the duration of patients’ response to this targeted therapy. BRCA testing is routinely performed in tumor tissue of ovarian cancer patients. The resulting molecular pathological findings include the genetic nomenclature of the mutation, the frequency of the mutated allele (variant allele frequency, VAF), and the tumor cell content. VAF measures the percentage of mutated alleles from the total alleles in the cells of the examined tissue. The aim of this study was to investigate the significance of VAF on the therapeutic response to PARPis in ovarian cancer patients. Epithelial ovarian cancer patients harboring BRCA1/2 tumor mutations, who underwent germline testing and received PARPi therapy at the Medical University of Vienna (*n* = 41) were included in the study. Corrected VAF (cVAF) was calculated based on VAF, tumor cell content, and germline mutation. Patients were divided into two groups based on their cVAF. Median PFS under PARPi in patients with low cVAF was 13.0 months (IQR [10.3-not reached]) and was not reached in the high cVAF group. High cVAF was significantly associated with longer PFS in the multivariate analysis (HR = 0.07; 95% CI [0.01–0.63]; *p* = 0.017). In conclusion, high cVAF was associated with a significantly better response to PARPi in this study population.

## 1. Introduction

Ovarian cancer is the second most lethal gynecological malignancy, following breast cancer, in western countries [1]. One of the predisposing factors for epithelial ovarian cancer (EOC) is germline mutation in tumor suppressor genes, such as BRCA1 and BRCA2 (BRCA1/2). These mutations can be inherited as a germline mutation or arise as somatic mutation in the tumor [2,3]. Germline BRCA1/2 mutations are found in approximately 18% of high grade serous ovarian cancer (HGSOC) patients [4,5], while exclusive somatic mutations in BRCA1/2 occur in about 3–8% [6,7].

During diagnostic workup, BRCA testing is performed on tumor tissue routinely in patients with histologically confirmed epithelial ovarian cancer. Patients with a tumor BRCA mutation are subsequently referred to genetic counseling and are advised to additionally perform germline BRCA testing. Germline testing is of clinical importance, as patients with germline BRCA mutation have an increased risk for breast cancer. Furthermore, relatives of patients with germline BRCA mutation could potentially carry the mutation themselves, increasing the risk for breast cancer, ovarian cancer in female and prostate cancer in male relatives.

BRCA testing is a standardized procedure performed routinely. The molecular diagnostic workup includes specific information about the respective gene mutation, allelic frequency of the detected mutation and percentage of tumor tissue (tumor cellularity or purity) in the examined tissue. Variant allele frequency (VAF) describes the “relative frequency of an allele at a genetic locus” which has to be corrected for the tumor cell fraction of the analyzed tissue (tumor cellularity) [8].

Poly (ADP-ribose) polymerase (PARP)-inhibitors (PARPi) as maintenance therapy following chemotherapy have increased treatment options for patients with BRCA1/2 mutation in recent years. Although PARPis have been shown to significantly improve the prognosis of this patient cohort, PARPi response rates depend on BRCA mutation status, therapy line and platinum sensitivity of recurrent disease and type of PARPi. Response rates for first-line maintenance PARPi treatment were found to be approximately 60–81% [9,10]. In recurrent disease, response rates of BRCA1/2 mutated patients differ between 69% for platinum sensitive tumors and 23% for platinum-refractory tumors [11]. The SOLO-3 trial found objective response rates of 72% for BRCA1/2 mutated patients with at least 2 prior lines of chemotherapy and 59% for patients with more prior therapy lines [12]. An integrated analysis of data from Study 10 and ARIEL2 revealed a response rate of 68% in patients with a BRCA1/2 mutation for third-line rucaparib treatment and 45% for fourth-line or later rucaparib treatment [13]. The possibility of PARPi resistance has led to extensive research to elucidate the underlying resistance mechanism [14,15]. To reduce potential severe side effects in patients with PARPi resistance and to optimize cost-effectiveness [16,17], it would be of interest to identify those patients, who would profit most of PARPi treatment. Although PARPis are usually well tolerated with common side effects including nausea (74–76%), fatigue (59–69%), vomiting (34–37%), and anemia (37–50%), mostly grade 1–2, higher grade and serious adverse events including hematological toxicities (anemia, neutropenia, thrombocytopenia), gastrointestinal toxicities (nausea, vomiting, abdominal pain, intestinal obstruction), fatigue, and rare secondary malignancies (myelodysplastic syndrome, acute myeloid leukemia) (0.83%) have been reported [18,19,20].

The significance of variant allele frequencies of somatic BRCA 1/2 mutations in epithelial ovarian cancer has not yet been entirely evaluated or associated with response to PARPi. Therefore, the aim of this study was to evaluate the effect of BRCA1/2 tumor allele frequency on PARPi response in a unicentric cohort of ovarian cancer patients.

## 2. Materials and Methods

### 2.1. Study Population

A total of 41 patients with EOC treated between 2014 and 2020 at the Medical University of Vienna (Comprehensive Cancer Center), Austria, were included in the study. The study was conducted according to the guidelines of the Declaration of Helsinki and approved by the Institutional Review Board of the Medical University of Vienna (2067/2019). All patients with tumor and germline BRCA1/2 mutation status, who received PARPi in primary or recurrent setting, were included in this study. Tumor samples of all included patients were routinely obtained during diagnostic or debulking surgery. BRCA tumor testing was performed at the Department of Pathology, Medical University of Vienna. Patients were further referred to genetic counselling and germline BRCA mutation assessment, at the Department of Obstetrics and Gynecology, Medical University of Vienna. All patients signed an informed consent for BRCA tumor testing prior to surgery.

Clinical data were retrieved from medical records, histological subtype and grading were determined by gynecologic pathologists. All patients were treated according to national guidelines and standard institutional procedures, consisting of either primary debulking surgery with adjuvant platinum-based chemotherapy +/− bevacizumab or neoadjuvant chemotherapy followed by intervention debulking surgery. Surgical staging was performed according to FIGO guidelines. PARPi therapy was initiated according to national approval and the respective tumorboard recommendation.

### 2.2. BRCA Germline Testing

Patients, who fulfilled the clinical criteria for germline testing, were referred to genetic counseling and gave written informed consent for BRCA germline testing/molecular analyses of a blood sample. BRCA germline testing was performed as previously described [21].

Genetic testing was performed at the University Hospital of Vienna from 2007 to 2015 with Sanger Sequencing in conjunction with multiplex ligation-dependent probe amplification (MLPA) and thereafter the Illumina TruSight Cancer panel on the MiSeq instrument was used for multigene panel testing according to the manufacturer’s instructions (Illumina, San Diego, CA, USA). Data were analyzed with the Sophia DDM^®^ software (Sophia Genetics, Boston, MA, USA)

### 2.3. BRCA Tumor Testing

BRCA tumor testing was performed as previously described [21]. Briefly, DNA was purified from FFPE tissue blocks with EZ1 DNA Tissue Kit (Qiagen, Hilden, Germany) on an EZ1 Advanced XL instrument (Qiagen). Next-generation sequencing libraries were generated with the Oncomine BRCA Research Assay (Thermo Fisher Scientific, Waltham, MA, USA) and sequenced with an Ion S5 system (Thermo Fisher Scientific). A threshold of 5% allele frequency was set for sequence variant calling. Variants were classified into pathogenic, likely pathogenic, variant of unknown significance (VUS), and likely benign/benign based on DNA sequence reference databases BRCA Exchange, ClinVar, COSMIC and dbSNP. The clinical report included only pathogenic/likely pathogenic sequence variants and VUS. To optimize data quality, only BRCA mutation reports with tumor cellularity greater than 10% were included in final analyses [6].

### 2.4. Calculation of Corrected Allele Frequency

Only the allele frequency of the tumor was of interest in this study; therefore, the specific corrected allele frequency of the tumor (*VAFt = cVAF*) had to be calculated from the overall allele frequency given in the molecular pathologic result (*VAF*). The given allele frequency is composed from the allele frequencies of the included tumor (*VAFt = cVAF*) and healthy (*VAFg*) tissues, according to the tumor cellularity (*Pt*) respective germline cellularity (*Pg* = 1 − *Pt*, as cellularity is given from 0 to 1).

VAF=VAFt×Pt+VAFg×Pg 

To correct the allele frequency for tumor purity and germline mutation status, the following formula, adapted from Kanchi et al. [22], was used:

$$cVAF=\frac{VAF-VAFg\times \left(1-Pt\right)}{Pt}$$

Germline mutations were only given as negative or positive results; therefore, *VAFg* was set as 0 in case of missing germline mutation or 0.5 (50% allele frequency) in case of detected BRCA germline mutation. cVAF was used for further analyses of treatment response.

### 2.5. Statistical Analysis

The corrected allele frequency (cVAF) was used for univariate and multiple Cox regression analyses. Time to progression under PARPi treatment (PFS) was defined as the time between start of PARPi treatment until tumor recurrence. Recurrence was confirmed radiologically according to RECIST 1.1 criteria. Patients who did not progress during follow up were censored respectively at the time point of last clinical contact. Median follow-up time of the cohort (only censored patients) was 30.0 months (interquartile range, IQR [15.9–37.3]). All statistical analyses were performed in GNU R 4.1.2 with following R-packages: survival 3.2-13, survMisc 0.5.5, and MASS 7.3-55. The optimal cut-off for a dichotomized cVAF predictor was determined by the cutp function from the R-package MASS and optically verified by a non-linear Cox regression modeling of the association between the cVAF and the hazard ratio for progression using smoothing splines, a penalized spline basis. Presented multiple Cox regression models were built by starting with the complete model (including all relevant clinicopathologic parameters) and selecting the most informative but smallest model by a backward elimination procedure minimizing the Akaike information criterion (AIC). To plot estimated survival curves for the optimally dichotomized cVAF from the final Cox-regression models, all correcting factors were averaged. For the predicted single patient survival curves, concrete values for the correcting factors were used. Continuous nominal values were compared between two groups with students’ *t*-test. Correlations of nominal variables with nominal or ordinal variables were performed using Spearman Correlations. Statistical significance was defined as *p*-value ≤ 0.05.

## 3. Results

### 3.1. Patient Characteristics

**Figure 1:**
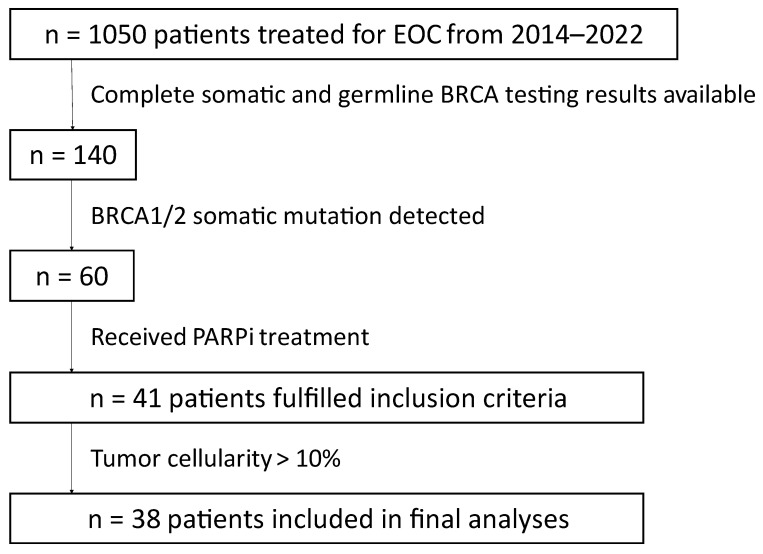
Scheme of patient selection. In total, 41 patients were included in the study and 38 patients were included in the final analyses.

A total of 41 patients with EOC fulfilled the inclusion criteria. Thereof, three patients were excluded from all further analyses due to ≤10% tumor cellularity, leaving 38 remaining patients for final analyses [6] (Figure 1).

Of these 38 patients, 35 patients had high-grade serous histology, three patients had high-grade endometrioid ovarian cancer. The vast majority of patients suffered from advanced stage FIGO III or IV ovarian cancer (*n* = 37, 97.4%), one patient had a FIGO stage II disease at initial diagnosis. Further patient characteristics are shown in Table 1.

Thirteen patients (34.2%) received neoadjuvant chemotherapy and intervention debulking surgery, 25 patients (65.8%) were treated by primary debulking surgery and adjuvant chemotherapy.

Median follow-up was 30.0 months (IQR [15.9–37.3]).

### 3.2. BRCA Testing

All patients underwent somatic and germline BRCA testing. With respect to tumor testing, 25 (66%) patients had a BRCA1 mutation and 13 (34%) patients had a BRCA2 mutation (34%) (Table 2). With respect to germline BRCA testing, 6 patients (15.8%) had no germline BRCA1/2 mutation, 22 (57.9%) a BRCA1, and 10 (26.3%) a BRCA2 mutation. There were no cases with multiple BRCA mutations. In 30 patients, tumor testing was performed on primary tissue and in eight patients, samples from recurrent tumors were tested. Of the nine patients receiving neoadjuvant chemotherapy with BRCA testing in primary tumor, four BRCA tests were performed on tissue obtained prior to chemotherapy, and five tests were performed on tissue obtained at intervention debulking after neoadjuvant chemotherapy.

Tumor cellularity ranged from 5% to 90%. Patients with ≤10% tumor cellularity were excluded from further analyses (*n* = 3) [6], resulting in 38 samples with 20% to 90% tumor cellularity.

Allele frequency evaluated in tumor tissue ranged from 6.4% to 89.8%, with 6.4% to 89.8% for germline BRCA1/2 wild-type patients compared to 49.9% to 88.8% for germline BRCA1/2 mutated patients. Corrected allele frequency (cVAF) ranged from 0.30 to 1.58, with a median of 0.60 [0.3;1.58] for germline wild-type and a median of 0.82 [0.56;1.27] for germline mutated patients. There was no significant difference in cVAF in patients with germline vs. wild-type BRCA mutation (*p* = 0.307) or in recurrent tumor samples compared to results obtained from primary tumor tissue (*p* = 0.155). (Figure S1). No significant correlations of cVAF with patients’ age or therapy-line were observed. (Figure S2).

### 3.3. PARPi Treatment

All patients received PARPi treatment, ranging from first-line to fifth-line therapy (Table 1). The vast majority (*n* = 11, 91.7%) of patients with first-line PARPi received bevacizumab prior to PARPi. In total, 32 (84.2%) patients received olaparib, four (10.5%) received niraparib, and two (5.3%) received rucaparib. Median duration of PARPi treatment was 12.94 months (IQR [9.6; 22.0]). Dose reduction was necessary in 12 (31.6%) patients and one (2.6%) patient discontinued PARPi treatment due to side effects. Of note, this patient developed acute myeloid leukemia (AML) and later died of that cause, while in complete remission of ovarian cancer. The majority of patients discontinued PARPi treatment due to progression (*n* = 19; 50.0%) and two (5.3%) due to other malignancy. A total of 16 (42.1%) patients were under PARPi treatment at time of censoring.

### 3.4. Progression-Free Survival

**Figure 2:**
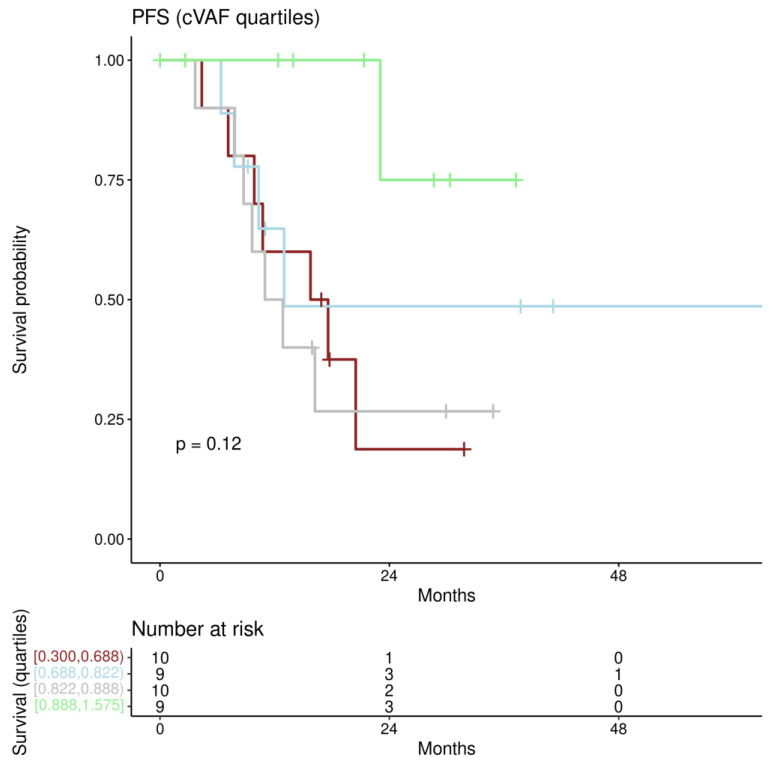
PFS for cVAF quartiles: Kaplan–Meier estimates for PFS categorizing cVAF along quartiles (Borders for cVAF are shown bottom left with “[” indicating including, and “)” indicating excluding). The lowest quartile is shown in brown, the second in blue, the third in grey, and the highest quartile in green.

PFS under PARPi ranged from 2.6 to 82.1 months, with 19 (50.0%) patients reaching the study endpoint (progression). For first univariate and multiple Cox regression analyses patients were stratified according to the quartiles of cVAF. Patients in the three lower quartile groups (0–25%, 25–50%, and 50–75%) showed median PFS of 16.7 months, 13.0 months, and 11.9 months, respectively, while the median PFS was not reached in the group with the highest (75–100%) cVAF (Figure 2).

**Figure 3:**
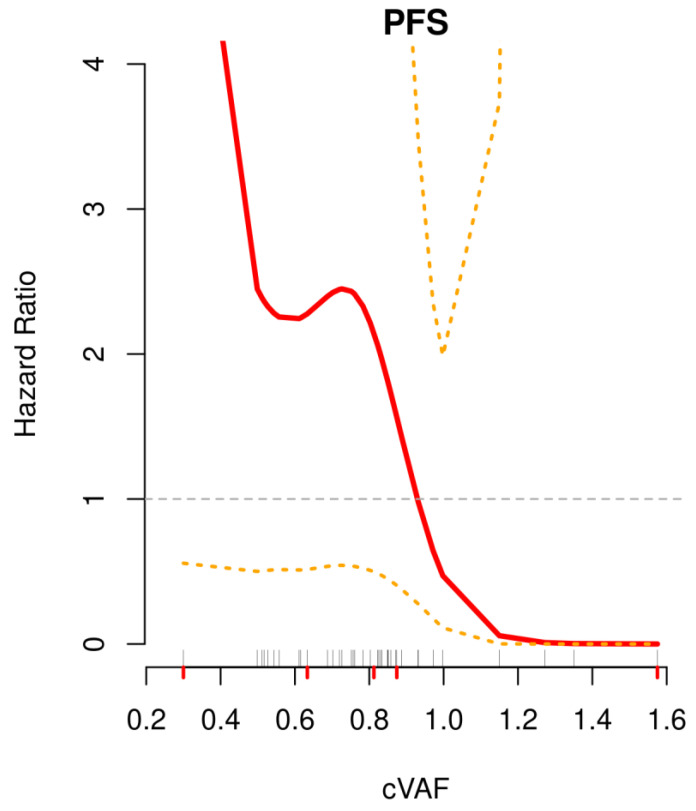
Non-linear modeling of the impact of cVAF on PFS using smoothing splines. Shown in red is the association of the hazard ratio with the cVAF with 95% confidence intervals (orange). Grey lines above the x-axis represent the single cVAF values, red lines below the x-axis the five-number summary (min, 25th percentile, median, 75th perc., max).

To determine the optimal cut-off of cVAF for PFS, the cutp function from R-package MASS was used, yielding 0.874 as the optimal cut-off with the lowest hazard ratio (HR) between the “high” (≥0.874; *n* = 10) and “low” (<0.874; *n* = 28) cVAF groups. To confirm this cut-off a non-linear modelling of the association of the cVAF value and the PFS was performed using smoothing splines, a penalized spline basis, shown in Figure 3. Here, the HR shows a steep decrease between 0.8 and 1.0, crossing the relative HR value of 1 by approx. 0.9 cVAF, confirming the optimal cut-off at 0.874.

Further analyses were performed using this cut-off for the separation in a cVAF low and cVAF high group.

The median PFS in the cVAF low group was 13.0 months (IQR [10.3-not reached]), while it was not reached in the cVAF high group.

**Figure 4:**
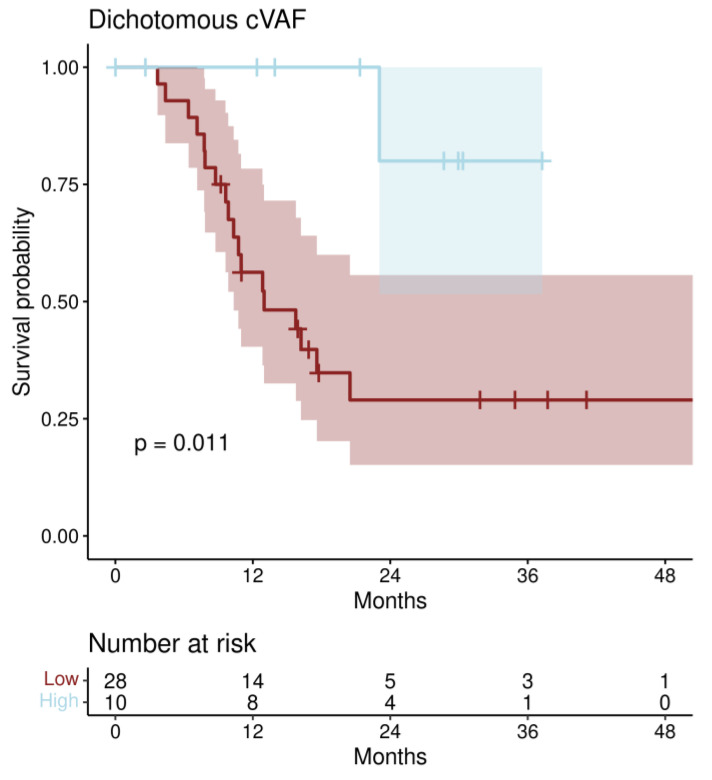
Kaplan–Meier estimates for PFS categorizing cVAF optimally in a low and high cVAF group. Shading areas are 95% confidence intervals.

**Figure 5:**
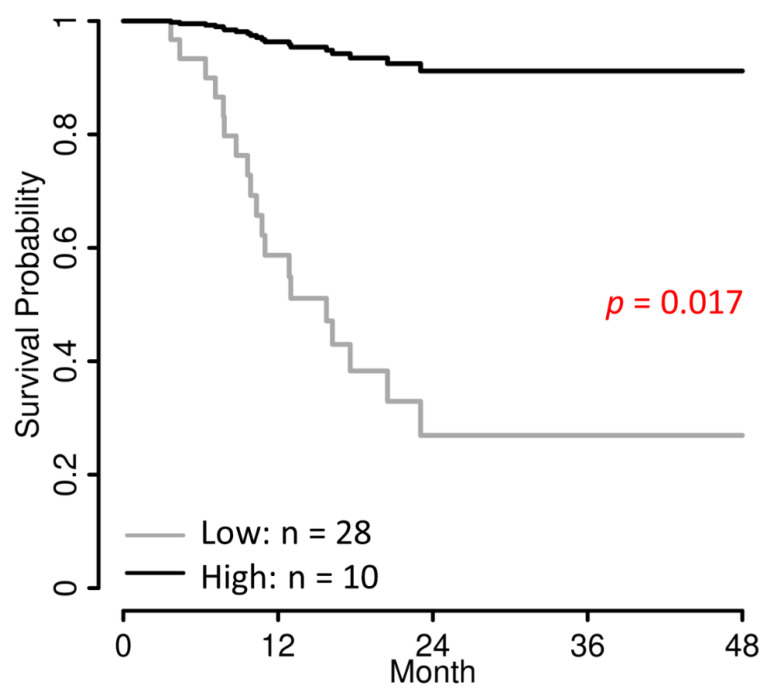
Progression free survival curves for low and high cVAFs according to the multiple Cox regression model (Table 3), including age as a correcting factor. As this survival curve is generated from a Cox regression model, no censored patients are indicated.

Univariate Cox regression analysis revealed a significantly association of the optimally dichotomized cVAF with PFS (HR 0.11; 95% CI [0.02–0.86]; *p* = 0.035) (Kaplan–Meier estimate, Figure 4). Using multiple Cox regression analysis starting with all relevant co-factors (age at start of PARPi treatment, FIGO stage at initial diagnosis, histology, BRCA mutation (BRCA 1 vs. 2), and the number of the PARPi treatment line) and selecting the minimal Cox-regression model by stepwise down-selection minimizing the AIC yielded a model comprising the two factors dichotomized cVAF (HR 0.07; 95% CI [0.01–0.63]; *p* = 0.017) and age. In Figure 5, survival curves of this model are shown and in Figure S3, a similar model (corrected for age) with the stratified cVAF quartiles is shown.

**Figure 6:**
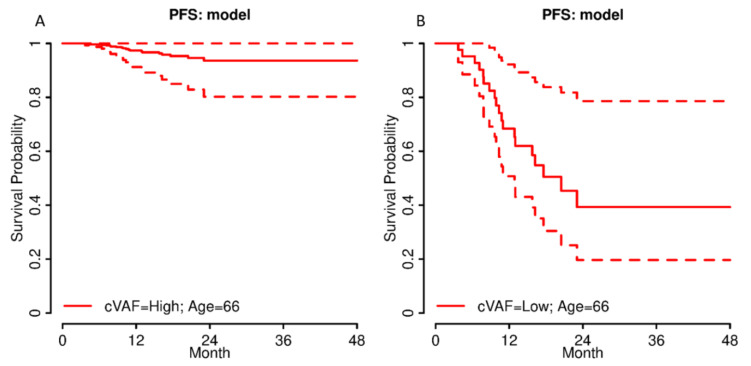
Survival curves with 95% confidence intervals according to the multiple Cox regression model from Table 3 for single patients with either a (**A**) high or (**B**) low cVAF (Table 3) and an age of 66 years.

Using the multiple Cox regression model, individual survival curves for every putative patient can be estimated. In Figure 6, exemplary survival curves with 95% confidence intervals for patients with age 66 and either high or low cVAF are shown.

A subgroup analysis of only HGSOC patients (*n* = 35) also revealed a significant impact of cVAF on PFS, univariately (HR 0.12; 95% CI [0.02–0.88]; *p* = 0.037) as well as corrected for age (HR 0.08; 95% CI [0.01–0.69]; *p* = 0.022).

There was no significant difference in PFS in patients with germline vs. somatic BRCA mutations (HR 0.82; 95% CI [0.23–2.86]; *p* = 0.754).

PARPi dose reduction was necessary in 1/10 patients in the cVAF high group and 11/28 in the cVAF low group. The one patient, who discontinued PARPi treatment and developed lethal AML, in complete remission of ovarian cancer, was in the cVAF high group.

## 4. Discussion

In this study, we showed for the first time that response to PARPi treatment was improved in EOC patients harboring a higher cVAF. The optimal cut-off point for the highest discrimination between both groups was by the 74% percentile, yielding 26% of patients with high cVAF values and a significantly better PFS after PARPi treatment in our study population (HR 0.07).

To date, data on targeted treatment response dependent on VAF are still scarce. The impact of VAF on patient survival was for example studied in lung cancer [23,24], oral squamous carcinoma [25], cervical cancer [26], and melanoma [27]. Friedlaender et al. recently showed a PFS benefit (but no overall survival benefit) for epithelial growth factor receptor (EGFR) mutated non-small cell lung cancer (NSCLC) patients treated with EGFR tyrosine kinase inhibitors (TKIs) with EGFR mutation allele frequency higher than 30%. Although their patient population was rather small (*n* = 31) and VAF was assessed from tumor biopsies, but not corrected for tumor cellularity, this is a hint for the importance of allele frequency in targeted treatment response. Comparable to our results, they found EGFR VAF and age >65 to be associated with PFS in a bivariable model [28].

Zheng et al. investigated the effect of the allele frequency of the T790M TKI resistance mutation in 54 NSCLC patients treated with osimertinib (the targeted treatment against T790M mutation). They did not identify an association of maximum somatic allele frequency (MSAF) with objective response rate (ORR) or PFS, but found a significant association of ORR and PFS with relative mutation purity (RPM), defined as the ratio of T790M VAF to MSAF. In this study, VAF was defined as the percentage of mutant DNA allele reads relative to total DNA allele reads (mutant and wild type). EGFR mutations and allele frequency were assessed with NGS of blood samples. Results were validated with an independent NSCLC cohort (*n* = 34) [29].

In melanoma, BRAF VAF was identified as a potential prognostic and predictive biomarker for treatment response to BRAF inhibitors. DNA of primary and recurrent tumor samples from 327 melanoma patients was used for identification of BRAF mutations. In total, 156 BRAF mutated patients were treated with BRAF inhibitor +/− anti-MEK inhibitor and patients with high VAF showed prolonged PFS and overall survival in multivariate analyses. VAF was defined as the “percentage of the mutated peak in the BRAF sequence” [30].

Stagni et al. discovered a worse PFS for melanoma patients with low BRAF AF treated with MAPK-inhibitors [27].

These studies, while performed in lung cancer and melanoma, are in line with our findings in ovarian cancer of improved benefit for targeted treatment in patients with higher VAFs/cVAFs of targeted mutations.

Previous studies assessed ORR or PFS under PARPi for patients with germline vs. somatic BRCA mutations. A meta-analysis by Mohyuddin et al. reported no significant difference in PARPi treatment response for patients with germline vs. somatic BRCA mutations in ovarian, breast, prostate, and pancreatic cancer. [31] In comparison, our study focusses solely on the amount of BRCA mutated alleles in the tumor cells, creating groups with high and low cVAF, both containing patients with germline and purely somatic BRCA mutations.

Dose reductions were necessary in 12 of 38 (31.6%) patients in our study, which is comparable to previously published data [18,32]. A higher rate of dose reductions was observed in the low cVAF group, potentially influencing treatment response rates.

This study has several potential limitations. A rather small cohort of 38 patients in a single academic center was included, limiting the generalizability of the reported results. Of the 26 patients treated with second to fifth line PARPi, 8 samples for BRCA testing were obtained from recurrent tumors, while 18 BRCA results stem from primary tumor samples, limiting the comparability. Furthermore, five somatic BRCA testings were performed on tissue obtained after neoadjuvant chemotherapy. It has been shown, that patients with BRCA1/2 mutated tumors undergoing platinum based chemotherapy can acquire secondary BRCA1/2 mutations, restoring BRCA function and, therefore, contributing to platinum and PARPi resistance [33,34,35]. These secondary BRCA mutations have not been assessed in our study and could influence the treatment response.

The possibility of newly arising BRCA1/2 mutations at recurrent disease can be excluded in our study cohort, as all patients where recurrent tumor was tested had germline mutations.

PARPi therapy line ranged from first to fifth line therapy with a majority of patients receiving second line-PARPi. Nevertheless, therapy line was included in our cox regression model and, therefore, results were corrected for PARPi therapy line. A substantially larger patient cohort would allow for a more uniformed-approach (e.g., for further studies) to only include certain/a single therapy line(s).

Only one sample per patient was used for somatic BRCA mutation testing; therefore, the possibility of the influence of intratumor heterogeneity on VAF and PARPi response remains.

Despite some limitations, this study represents a rather big and uniform cohort of patients all tested and treated at one single institution. Considering that PARPi treatment is a relatively new therapy approved for EOC patients, the patient number is appropriate. Patients underwent regular follow-up visits, granting good data quality and comprehensive follow-up.

For further investigation of the influence of cVAF on treatment response to PARPi, larger (multi-center) studies with bigger patient cohorts and more uniformed criteria (such as only first or second line PARPi) would be needed. A comparison of low cVAF patients to non-BRCA1/2mut patients receiving PARPi treatment would be of interest.

## 5. Conclusions

We showed, for the first time, a tremendously improved progression free survival time for 26% of PARPi treated epithelial ovarian cancer patients (with one to four preceding chemotherapy lines) with high corrected variant allele frequencies (cVAF) of either a BRCA1 or a BRCA2 mutation. Age was the only confounding factor, significantly improving the progression free survival (PFS) Cox regression model.

## Figures and Tables

**Table 1:** Patient characteristics at primary diagnosis of 38 patients with tumor cellularity >10%.

Parameter	Variable	*n* (%)
Histology	Serous	35 (92.1%)
	Endometrioid	3 (7.9%)
Grade	high-grade	38 (100.0%)
FIGO stage at initial diagnosis	II	1 (2.6%)
	III	27 (71.1%)
	IV	10 (26.3%)
Primary treatment	NACT + IDS	13 (34.2%)
	PDS + adj. CHT	25 (65.8%)
PARPi	Olaparib	32 (84.2%)
	Niraparib	4 (10.5%)
	Rucaparib	2 (5.3%)
PARPi Treatment line	1	12 (31.6%)
	2	20 (52.6%)
	3	1 (2.6%)
	4	3 (7.9%)
	5	2 (5.3%)

**Table 2:** BRCA mutation results.

		Germline Mutation		Germline Wild-Type
		BRCA1	BRCA2	
**Somatic mutation**	BRCA1 (*n* = 25)	22	0	3
	BRCA2 (*n* = 13)	0	10	3
		22	10	6

**Table 3:** Univariate and multiple Cox-Regression for progression-free survival.

Parameters (*n* = 38, 19 Events)	Univariate HR [95% CI]	*p*-Value	Multiple HR [95% CI]	*p*-Value
cVAF	0.11 [0.02–0.86]	0.035	0.07 [0.01–0.63]	0.017
Age ^1^	0.99 [0.96–1.03]	0.733	0.96 [0.91–1.01]	0.145
Therapy-line	1.21 [0.83–1.78]	0.327	- ^4^	-
FIGO at initial diagnosis ^2^	1.02 [0.37–2.86]	0.964	- ^4^	-
Histology ^3^	0.43 [0.05–3.49]	0.428	- ^4^	-

^1^ Age at start of PARPi treatment. ^2^ FIGO: stage IV (metastatic) vs. stage II/III at initial diagnosis. ^3^ Histology: serous vs. endometrioid. ^4^ removed from the model by step-wise down-selection, minimizing the Akaike Information Criterion (AIC).

## Data Availability

The data presented in this study are available upon reasonable request from the corresponding author. The data are not publicly available due to privacy and ethical reasons.

## Supplementary Materials

The following supporting information can be downloaded at: https://www.mdpi.com/article/10.3390/jpm12091467/s1, Figure S1. Boxplot of cVAF in primary and recurrent tumor tissue; Figure S2: Correlation coefficient matrix for cVAF, age and therapy line; Figure S3: Progression free survival curves for cVAFs quartiles according to the multiple Cox regression model.
