# Supplementary material for: Corrected Allele Frequency of BRCA1/2 Mutations Is an Independent Prognostic Factor for Treatment Response to PARP-Inhibitors in Ovarian Cancer Patients
Source: J Pers Med. 2022 Sep 7;12(9):1467. doi: 10.3390/jpm12091467 (PMC9504000; doi:10.3390/jpm12091467)
Supplement: Supplementary file 1 [file jpm-12-01467-s001.zip › jpm-1885526-supplementary.pdf]

Corrected Allele Frequency of BRCA1/2 Mutations Is an Independent Prognostic Factor for Treatment Response to PARP-Inhibitors in Ovarian Cancer Patients

Christina T. Grech [1,2], Dietmar Pils [2], Stefanie Aust [1,*], Christoph Grimm [1], Stephan Polterauer [1], Alexander Reinthaller [1], Leonhard Müllauer [3], Theresa Reischer [1] and Christine Bekos [1]

[1] Department of Obstetrics and Gynecology, Division of General Gynecology and Gynecologic Oncology, Gynecologic Cancer Unit, Comprehensive Cancer Center (CCC), Medical University of Vienna, 1090 Vienna, Austria
[2] Department of General Surgery, Division of Visceral Surgery, Comprehensive Cancer Center (CCC) Vienna, Medical University of Vienna, 1090 Vienna, Austria
[3] Department of Pathology, Medical University of Vienna, 1090 Vienna, Austria
* Correspondence: stefanie.aust@meduniwien.ac.at; Tel.: +43-1-40400-29150

Supplementary Material

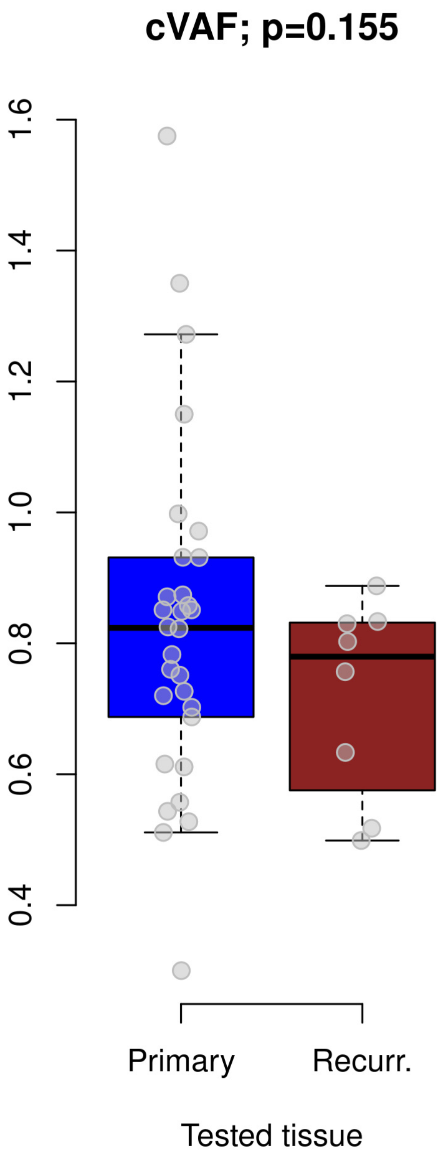

Supplementary Figure S1. Boxplot of cVAF in primary and recurrent tumor tissue, showing no significant difference.

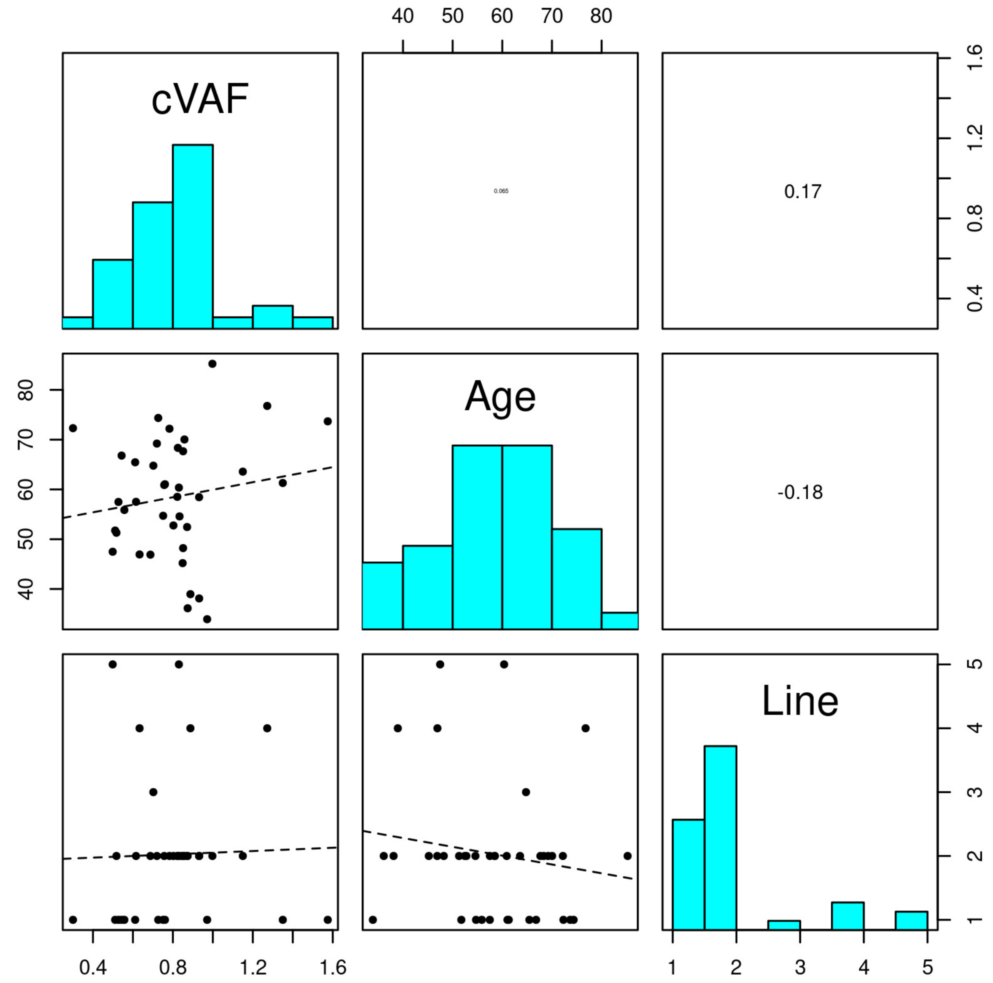

Supplementary Figure S2. Correlation coefficient matrix for cVAF, age and therapy line. The top left, middle and bottom right quadrants show histograms of the distribution of the values. X-axis for the left and right quadrants is shown at the bottom and for the middle quadrants at the top of the panels. Lower left quadrants show scatterplots. Higher right quadrants show correlation coefficients (font size correlates with value, upper middle coefficient is 0.065). No significant correlations were observed.

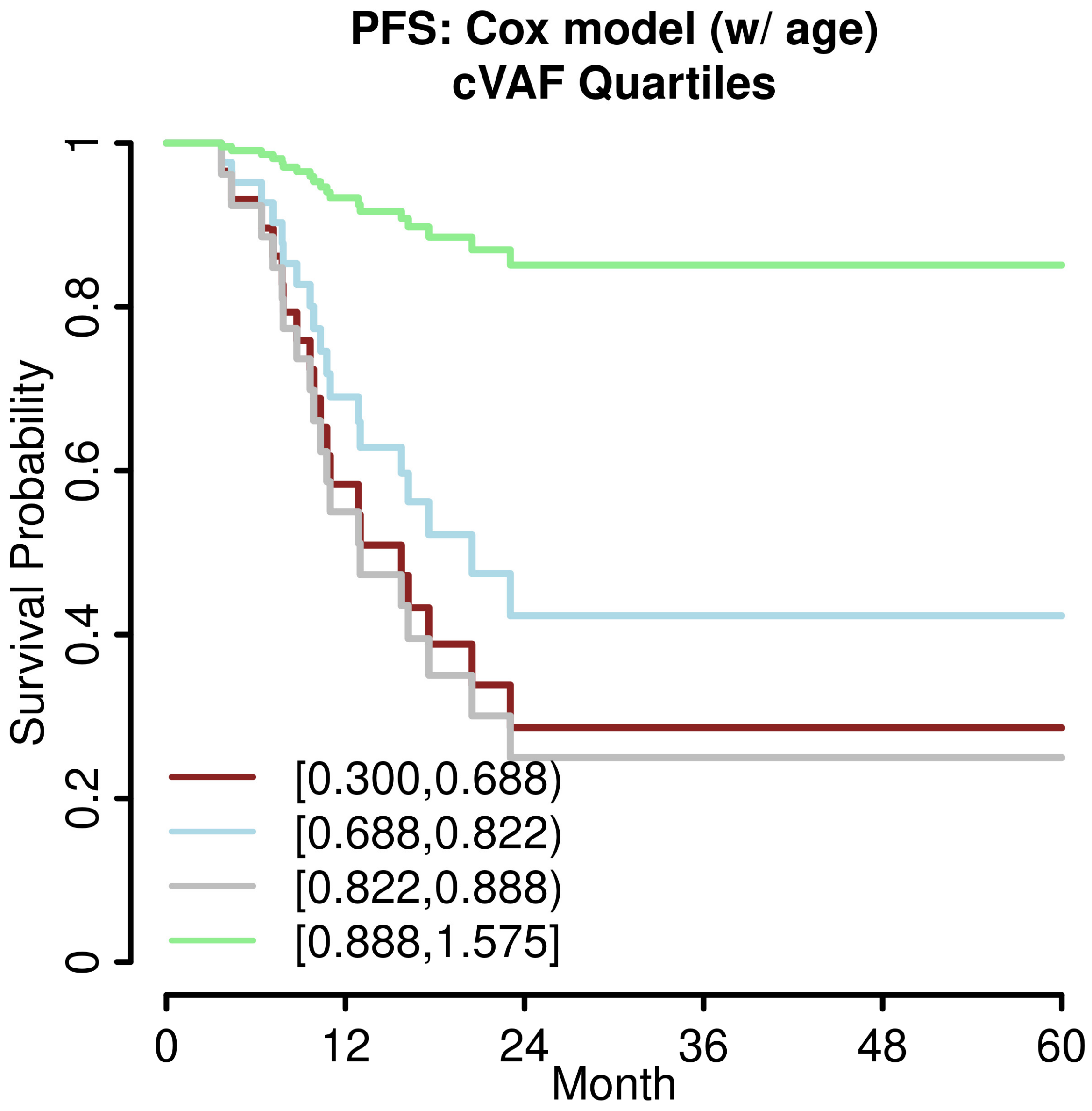

Supplementary Figure S3. Progression free survival curves for cVAFs quartiles according the multiple Cox regression model, including age as a correcting factor. Borders for cVAF are shown bottom left with "[" indicating including and ")" indicating excluding. As this survival curve is generated from a Cox regression model, no censored patients are indicated.
